# A compulsory pop-up form reduces the number of vitamin D requests from general practitioners by 25 percent

**DOI:** 10.1080/02813432.2020.1794399

**Published:** 2020-07-20

**Authors:** Jens K. Munk, Lise Bathum, Henrik L. Jørgensen, Bent S. Lind

**Affiliations:** a Department of Clinical Biochemistry, Hvidovre University Hospital, Hvidovre, Denmark; b Department of Clinical Medicine, University of Copenhagen, Copenhagen, Denmark

**Keywords:** Vitamin D, healthcare cost, evidence-based interventions, general practitioner, laboratory testing

## Abstract

**Objective:**

Healthcare costs, including costs for laboratory tests, are increasing worldwide. One example is the measurement of vitamin D. General practitioners in the Capital Region of Denmark include a vitamin D status in approximately 20% of all laboratory requisitions. This study intended to examine the effect of a compulsory pop-up form in the electronic request system on the number of vitamin D tests and to monitor the indications.

**Design:**

From 1 January 2017, we introduced a compulsory pop-up form in which the general practitioners had to state the indication for measuring vitamin D, choosing from a predefined set of indications. Intervention practitioners were compared with control practitioners before and after the intervention.

**Setting:**

General practices in the Capital Region of Denmark.

**Subjects:**

In total, 572 general practitioners and 383,964 patients were included in the period from 1 January 2016 to 31 December 2018.

**Main outcome measures:**

Number of vitamin D tests and distribution of indications.

**Results:**

We observed a drop in number of vitamin D requisitions to 70% (in 2017) and 75% (in 2018) relative to 2016. During the same period, the number of requisitions increased by 33% in a non-intervention group of practitioners. The indication ‘Monitoring of treatment with vitamin D’ was the most frequently used indication, recorded in 121,475 patients.

**Conclusion:**

A compulsory pop-up form reduces the number of vitamin D requests from general practitioners by 25%. The implication is that pop-up forms can be used to decrease healthcare costs.

## Introduction

Danish forecasts for the economic consequences of population ageing and longer life expectancy predict that more resources must be allocated to healthcare if we are to maintain the current standards. The cost increase is estimated to be more than 2% every year for the next 10 years [1]. This development makes it imperative to find new ways to diminish unnecessary health costs.

Over the last decades, several studies have described efforts to improve appropriate use of laboratory tests [2,3] in hospital settings [4–8] as well as in primary healthcare [9,10]. Most studies describe the effects of different interventions at the laboratory level to reduce laboratory test requesting. These interventions are classified as weak, moderate or strong, depending on the power of their impact on the reduction of inappropriate testing [11]. Educational-based approaches, such as referring to evidence-based guidelines, are considered weak tools that need to be coupled with other interventions. The moderate and strong tools are usually incorporated into the laboratory, hospital and/or primary healthcare information systems. They include various restrictions in the requesting process, such as a limitation in or a complete removal of test availability, requesting algorithms and reflex testing, but also request entry design including decision support and the use of pop-ups.

Vitamin D is a hormone known to be essential for bone health. Low levels of vitamin D are furthermore considered a risk factor for several diseases such as cardiovascular disease, cancer, diabetes, autoimmune disease and infections [12]. The multifaceted involvement of vitamin D in physiological processes has prompted physicians and patients to demand more vitamin D testing, increasing the number of tests exponentially [4,13]. In our part of the Capital Region of Denmark, representing 20% of the nation’s inhabitants, vitamin D testing requested by general practitioners increased from approximately 1 in 500 inhabitants in 2004 to 1 in 5 inhabitants in 2010 [13]. This very large increase in the number of vitamin D tests raised the question of potential overuse and called for action.

There is an ongoing debate as to who should be tested for vitamin D deficiency. While vitamin D deficiency is frequent, the testing is expensive, and universal testing is not recommended [14]. No high or moderate quality evidence has been found to support the effect of vitamin D on non-bone health outcomes other than falls [14]. Vitamin D testing is considered beneficial for patients at risk of severe deficiency such as patients with insufficient dietary vitamin D intake or limited exposure to sunlight, or patients with gastrointestinal malabsorption or renal disease [15].

In 2010, the Danish Health Authority imposed a weak intervention by publishing recommendations regarding prevention, diagnosis and treatment of vitamin D testing [16], and the vitamin D testing temporarily decreased by 15% to 1 in 6 inhabitants in 2011 [17]. Five main indications for the measurement of vitamin D were stated in the recommendation. These indications were used to introduce a compulsory pop-up form in the general practitioners electronic request system from January 2017 in the Capital Region of Denmark where the general practitioners had to state the indication for the vitamin D test.

The aim of the present study is to describe the effect of this compulsory pop-up form and moreover, we aim to describe the distribution among the different indications.

## Materials and methods

### Patients

Patients were included from all general practitioners in the Capital Region of Denmark, who were active in all three years defined as having requested at least one vitamin D test per calendar year in 2016, 2017, and 2018.

### Data

Vitamin D data from 1 January 2016 to 31 December 2018 were extracted from the Laboratory Information System (Labka II, CSC Denmark) from the laboratories in the Capital Region of Denmark serving the general practitioners in the region. Patient age and sex were extracted from the data, after which patients were anonymised by assigning a running number unique to each individual. The dataset from the three years contained a total of 633,026 vitamin D measurements requested by general practitioners in the Capital Region. Of these, 608,708 vitamin D measurements were requested by practitioners that were active throughout the study period. Non-numeric results containing ‘>’ were set to 526 nmol/L, results containing ‘<’ were set to 0. All other non-numerical results (*N* = 7115), measurements above 526 nmol/L (*N* = 6), and measurements with unintelligible indications (*N* = 6), were excluded. The remaining 601,581 measurements were requested by general practitioners on the island of Bornholm (*N* = 9637 from 6113 patients and 11 general practitioners) where the intervention was not implemented and by general practitioners in the rest of the Capital Region (*N* = 591,944 from 377,851 patients and 561 general practitioners). The latter is denoted the intervention group and Bornholm the non-intervention group.

### Measurement of vitamin D

Vitamin D was measured in blood by commercially available immunoassays at the 6 laboratories analysing samples requested from general practitioners. The assays were from Siemens, Roche, Beckman Coulter or DiaSorin and they measured the sum of 25-hydroxyvitamin D2 and D3. None of the laboratories changed methods during the 3-year study period. All laboratories participated in external quality control programs.

### Intervention and indications for vitamin D measurement

Based on the recommendation from the Danish Health Authority [16], starting 1 January 2017 we imposed a moderate intervention by requiring the general practitioners to choose one of six possible indications for requesting a vitamin D measurement. These six indications were 1) ‘Low sun exposure/veiling’, 2) ‘Monitoring of treatment with vitamin D’, 3) ‘Skeletal pain, osteoporosis, neural or muscular symptoms’, 4) ‘Hyperparathyroidism, hyper- or hypocalcemia’, 5) ‘Gastrointestinal malabsorption’, and 6) ‘Other’. These six indications were shown in a pop-up window in the electronic request system used by the general practitioners (WebReq) when a vitamin D test was requested. When ‘Other’ was chosen, the general practitioner had to write the indication. No verification of the chosen indications was performed. The intervention was not implemented on the island of Bornholm, which was therefore used as a control group.

To prevent negative reactions, the intervention was implemented after discussion and agreement between the laboratories and representatives of the general practitioners as well as of the local health authorities. Furthermore, in December 2016, a detailed information about the intervention was sent to all general practitioners in the Capital Region.

### Statistical analysis

All statistical analyses were performed using the statistical tool R [18]. Student’s *t* test was used to establish whether two samples differed significantly. The percentage of all requests that included a vitamin D status was calculated for each general practitioner before and after the intervention.

## Results

Table 1 summarizes the basic characteristics of the patients in the intervention group and the non-intervention group in 2016. In the non-intervention group, there was a greater percentage of female patients, the patients were older (*p* ≪ 0.05, t test) and they had lower vitamin D levels (*p* ≪ 0.05, t test).

**Table 1.** Characteristics of patients and vitamin D measurements in the intervention group and the non-intervention group in 2016, prior to intervention.

Parameter	Intervention group	Non-intervention group
Number of vitamin D measurements (N)	241,424	2789
Females, %	64.6	72.7
Median vitamin D, nmol/L (IQR)	77 (43)	65 (39)
Median age, years (IQR)	53.6 (32.2)	58.5 (26.6)
The 3 months of lowest vitamin D level, % of N^a^	20.7	21.8
The 3 months of highest vitamin D level, % of N^a^	27.0	25.3
Results between 25 and 50 nmol/L, % of N	15.2	24.8
Results between 12 and 25 nmol/L, % of N	3.4	3.3
Results below 12 nmol/L, % of N	0.59	0.04

IQR: interquartile range.

^a^The seasonal variation in vitamin D level was associated with a higher number of vitamin D requests during the three months where the median vitamin D level was highest (June 29 to September 26) compared to the three months where it was lowest (January 25 to April 23).

Table 2 describes the study population from the general practitioners where the intervention was imposed. We observed a decrease in the number of vitamin D measurements of 30% from 2016 (*N* = 241,424) to 2017 (*N* = 169,775) and of 25% from 2016 to 2018 (*N* = 180,745). In comparison, the non-intervention group showed an increase in the number of vitamin D measurements of 12% from 2016 (*N* = 2789) to 2017 (*N* = 3130) and of 33% from 2016 to 2018 (*N* = 3718). The median vitamin D level decreased significantly (*p* ≪ 0.05, t test) from 77 nmol/L to 71 nmol/L after the implementation of the intervention. Furthermore, median age dropped significantly from 53.6 years to 52.4 years (*p* ≪ 0.05, t test).

**Table 2.** Characteristics of patients and vitamin D measurement in the intervention group from 2016 through 2018.

	2016	2017	2018
Vitamin D measurements N (index^a^)	241,424 (100)	169,775 (70)	180,745 (75)
% Females	64.6	66.8	66.5
Median age, years (IQR)	53.6 (32.2)	51.9 (32.6)	52.4 (33.1)
Median vitamin D, nmol/L (IQR)	77 (43)	71 (42)	71 (43)

^a^The index is shown using 2016 as baseline.

IQR: interquartile range.

For each year, repeated measurements were calculated (Table 3). Approximately 15% of all measurements were repetitions on the same patients within the same calendar year. There were up to 14 measurements on the same patient within one calendar year, and up to 26 measurements per patient over the course of the three years. Following the intervention, the reduction in measurement counts was more pronounced for patients with multiple measurements than for patients with a single measurement.

**Table 3.** Number of patients with 1, 2–4 or more than 4 vitamin D measurements per year, and number of measurements performed on these patient groups.

	Number of patients (index^a^) [number of measurements]		
	2016	2017	2018
One measurement	173,258 (100) [173,258]	124,584 (72) [124,584]	133,211 (77) [133,211]
2–4 measurements	30,085 (100) [66,528]	20,171 (67) [44,258]	21,261 (71) [46,544 ]
>4 measurements	92 (100) [633]	47 (51) [348]	51 (55) [345]

^a^The index is shown using 2016 as baseline. Indexes show that the drop in measurement counts is more pronounced in patients with multiple measurements than in patients with a single measurement. Number of analyses are given in square brackets.

**Figure 1.**
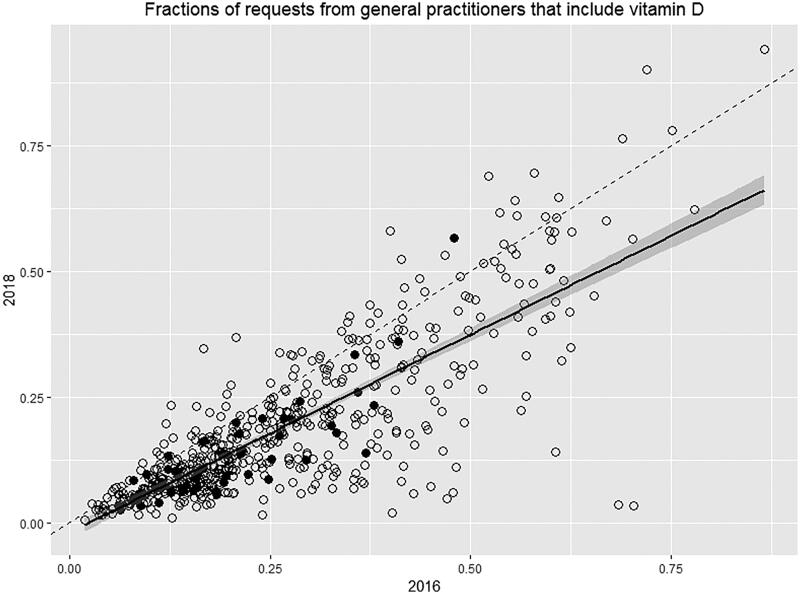
Correlation between the fraction of request forms from general practitioners with a request of vitamin D in 2016 (x-axis) compared with 2018 (y-axis). Each symbol is one general practitioner. Solid symbols represent the 10% of general practitioners with the highest total number of requisitions. The dashed line represents the line of no change. The solid line represents the correlation between 2016 and 2018. The grey area represents ± 1 SD.

In total, the 561 general practitioners in the intervention group accounted for 591,944 measurements. In 2018, the 10% most active of these general practitioners requested 31% of the vitamin D measurements, while the 25% least active of these general practitioners only requested 6% of the vitamin D measurements. For all 561 general practitioners, we calculated the fraction of all requests that included vitamin D each year. The correlation between these fractions for 2016 and 2018 is shown in Figure 1. Allowing for a run-in period, the fractions for 2017 were not used. We observed a correlation between the fraction of requisitions containing a vitamin D before and after the intervention for each general practitioner. Also, most of these general practitioners lay below the dashed line, showing that their fraction was reduced from 2016 to 2018. The fractions of vitamin D requests varied widely as some of these general practitioners only rarely included a vitamin D measurement and others included a vitamin D measurement in most request forms. Finally, Figure 1 shows that the general practitioners requesting the most samples have the lowest fraction of requests containing a vitamin D measurement.

**Figure 2.**
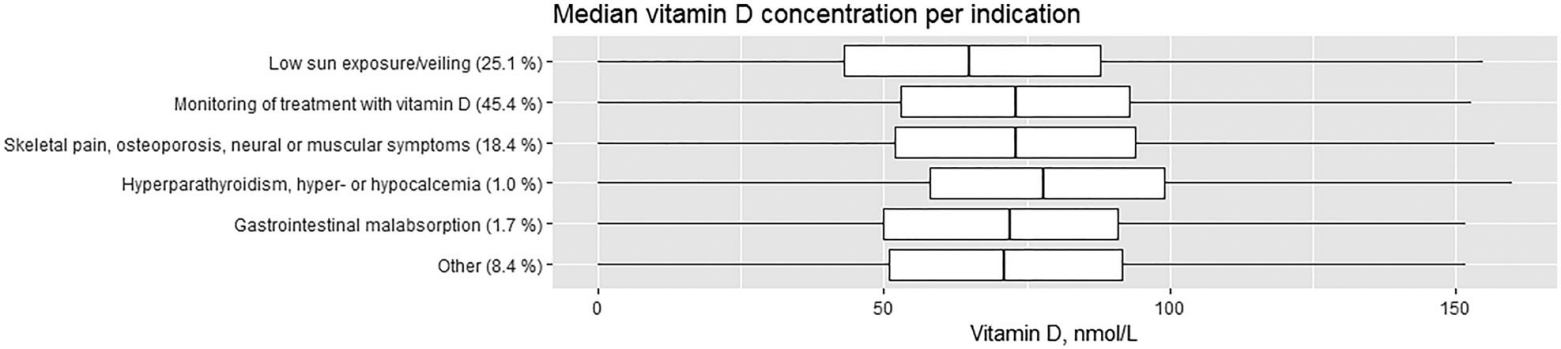
Box plot showing the median vitamin D concentration as well as the percentage of requests for each of the six indications. The level of vitamin D is recommended to be >50 nmol/L (Ref. [16]). The number of indications was 350,520 from both 2017 and 2018.

Figure 2 shows the stated indications for the vitamin D measurement and the median vitamin D levels for each of the six indications. For each indication, the percentage of measurements was stated. Of the 350,520 measurements requested by general practitioners in the intervention group in 2017 and 2018, 2166 were removed because they did not have an indication assigned. The lack of indications was due to ordering prior to the implementation of the intervention with blood sampling after the implantation of the intervention. The indication ‘Other’ had to be followed by a written indication. Among the 29,259 vitamin D requests in this group, 15% had stated ‘fatigue’ as the indication, making this the most frequent indication in this group. A visual inspection of the distribution of requests in the 6 indication groups showed no difference in ordering frequency throughout the year (data not shown).

The indication ‘Monitoring of treatment with vitamin D’ was the most frequently used indication, recorded in 121,475 patients. Of these, 26,472 had between two and 14 recorded measurements, 93,615 measurements in total. The time difference between two consecutive measurements was calculated and the following pattern in follow-up measurements was observed: 10% of follow-up were made within the first two months. During months 3 and 4 after the initial measurement, 22% of the follow-up measurements were made. Between months 5 and 10, 41% of follow-up were made, and another 20% of follow-up were made between months 11 and 15, The remaining 7% were made more than 15 months after the previous measurement.

## Discussion

### Principal finding

The current study is the first of this magnitude to describe the consequences of implementing a compulsory pop-up form in which the general practitioners had to state the indication for measuring vitamin D. We observed a 25% decrease in the number of vitamin D requests after implementing this intervention. On the other hand, there was a 33% increase in vitamin D requests from general practitioners in the non-intervention group. These observations strongly support that the intervention is the main reason for the reduction in the number of vitamin D requests.

The median vitamin D concentration decreased after implementation of the intervention, which could indicate that the intervention did indeed reduce unnecessary measurements on healthy individuals. From the large number of requests, it seems reasonable to assume that many vitamin D requests are not based on the official recommendations from the Danish Health Authority. Throughout the entire study period, more than one-quarter of the measurements were repetitions within one year. A few patients had up to 14 measurements within one year, which is far beyond any recommendations by the health authorities [16].

Furthermore, both before and after the implementation, we observe a pronounced variation in vitamin D ordering pattern with many of the general practitioners having only a few percent of requests including vitamin D and others having more than 80% including vitamin D (Figure 1). Furthermore, as can be seen in Table 1, most measurements were in fact performed during the summer period where the vitamin D status is highest, and a measurement should not be necessary. Such variation in test ordering pattern again raises the question of possible overuse and highlights the potential for future interventions to further reduce the number of unnecessary tests [2].

Figure 2 shows that in 92% of the indications for ordering vitamin D, the general practitioners chose one of the indications from the national recommendations [16]. The indication ‘Other’ was included in the intervention because the national recommendation was considered too restrictive by some general practitioners. In 8% of the total number of indications ‘Other’ was chosen. The indications stated in this group were very diverse making it difficult to discern a pattern. The most common indication stated was ‘fatigue’. Nevertheless, unspecific fatigue is not an indication for a vitamin D measurement according to the national guidelines [16].

### Strengths and weaknesses of the study

The major strengths of this study are the sample size and the setting in primary healthcare, where the initial suspicion of vitamin D deficiency usually arises. Furthermore, the data in the study are real life data covering three years of clinical practice. Another strength of the study is that the indications for vitamin D testing in the pop-up window were defined in accordance with the national recommendations. The reduction in the number of vitamin D tests after implementation of the intervention indirectly supports the suspicion of inappropriate overuse of vitamin D testing before the intervention was implemented.

One possible weakness could be that we had a small non-intervention group which, as can be seen in Table 1, was different from the intervention group especially regarding patient age and sex distribution. However, the non-intervention group had a major increase in number of requisitions. This could potentially result in an underestimation of the effect and does not alter the conclusion.

### Findings in relation to other studies

Our results substantiate the results of previous studies on changing test ordering patterns in primary healthcare [9–11] while expanding the knowledge on how to reduce laboratory test ordering by changing the ordering procedure. The study demonstrates how a guideline from the health authorities on vitamin D testing, which can be regarded as a weak tool, can be combined with an intervention regarded as a moderately strong tool to reduce the number of vitamin D tests [11], thereby supporting a more appropriate use of laboratory tests. From a health cost point of view, this is of high importance.

### Meaning of the study

In this paper, we describe a general intervention that reduced the amount of vitamin D requests from general practitioners by 25%. We analysed the ordering pattern before and after the intervention as well as the indications chosen by the general practitioners for ordering the vitamin D measurement. The general intervention described in this study should probably be followed by a more specific intervention aimed at those general practitioners requesting the largest number of vitamin D measurements. Possible interventions could be a time limit between measurements or a personal visit by laboratory representatives to the general practitioners with the highest number of vitamin D requests.
